# Does Physical Activity Reduce the Risk of Perceived Negative Health in the Smoking Population?

**DOI:** 10.3390/ijerph192114366

**Published:** 2022-11-03

**Authors:** Ángel Denche-Zamorano, David Manuel Mendoza-Muñoz, Damián Pereira-Payo, Manuel J. Ruiz, Nicolás Contreras-Barraza, José A. Iturra-González, Javier Urbano-Mairena, Carolina Cornejo-Orellana, María Mendoza-Muñoz

**Affiliations:** 1 Promoting a Healthy Society Research Group (PHeSO), Faculty of Sport Sciences, University of Extremadura, 10003 Caceres, Spain; 2 Promoting a Healthy Society Research Group (PHeSO), Department of Psychology and Anthropology, Education and Psychology Faculty, University of Extremadura, 06006 Badajoz, Spain; 3 Facultad de Economía y Negocios, Universidad Andres Bello, Viña del Mar 2531015, Chile; 4 Escuela de Medicina, Facultad de Ciencias Médicas, Universidad de Santiago de Chile (USACH), Santiago 9170022, Chile; 5 Escuela de Negocios y Tecnología, Universidad Gabriela Mistral, Santiago 7500533, Chile; 6 Research Group on Physical and Health Literacy and Health-Related Quality of Life (PHYQOL), Faculty of Sport Sciences, University of Extremadura, 10003 Caceres, Spain; 7 Departamento de Desporto e Saúde, Escola de Saúde e Desenvolvimento Humano, Universidade de Évora, 7004-516 Évora, Portugal

**Keywords:** health, self-perceived health, smoking, odds ratio, physical activity

## Abstract

Background: Smoking is associated with poor health status. Increased prevalence of multiple diseases has been found in populations of smokers and ex-smokers. Physical activity (PA) could reduce the negative effects of smoking. Aims: To analyze the relationships between smoking and self-perceived health and between PA level and self-perceived health, according to the relationship with smoking in the Spanish population. To calculate the risks of perceiving negative health in relation to smoking, according to the PA level of the population. Hypothesis: A higher level of PA reduces the risk of perceiving negative health in the Spanish smoking population. Design and Methodology: Cross-sectional study with data from 17,708 participants, 15–69 years old, interviewed in the Spanish National Health Survey 2017. Intergroup differences were studied. Odds ratios (OR) and relative risks (RR) and their confidence intervals (95% CI) were calculated for negative self-perceived health. A Spearman’s *rho* correlation study was performed between the variables of interest. Results: Dependency relationships were found between self-perceived health and PA levels, in both genders and in different relationships with smoking (x^2^ < 0.001). Inactivity was related to higher prevalences of negative health perception (*p* < 0.05) in all groups analyzed. Inactive smokers (OR: 6.02. 95% CI: 3.99–9.07. RR: 5.24. 95% CI: 3.56–7.73) presented increased risks of negative health perception compared to people with low/medium PA levels, similarly found in other relationships with tobacco. Conclusions: Increasing the PA level of the smoking population could reduce the negative effects on their perceived health. Medium and high PA levels reduce the risk of negative health perception in the Spanish population, both in smokers, ex-smokers, and non-smokers.

## 1. Introduction

In Spain, there has been a reduction in the proportion of smokers in recent decades, with the prevalence of tobacco use in the Spanish population falling from a third to a quarter of the population from the early 1990s to the present [1]. Despite this, annual deaths attributable to tobacco use are estimated to be more than 50,000 people nationwide [2], being the leading cause of preventable premature death in the world and being associated with numerous causes of death [3,4].

Smoking has been associated with a large number of pathological conditions. It is associated with an increased risk of coronary artery disease, increased risk of atherosclerosis, associated with 17 types of cancers, mental illness, pulmonary fibrosis, and chronic obstructive pulmonary disease [5,6], among other pathologies. Quitting smoking, or reducing tobacco consumption, has been shown to be beneficial to health, as well as to reduce the risk of lung cancer and the likelihood of cardiovascular disease in cases of strong reductions in consumption, although it may not be a sufficient condition to reduce all-cause mortality [7]. In addition, smokers and ex-smokers have been found to have decreased cardiorespiratory [8,9], pulmonary [10], and functional [11,12] capacities. A reduction in global and mental self-perceived health (SPH) was also shown, being one of the reasons for smoking cessation [13,14].

The SPH, regardless of the relationship with smoking, is a factor to be taken into account in the population, being widely used, given its reliability and reliability to assess the health of people in a quick and simple way, obtaining relevant information on the health status of the population through interviews or questionnaires [15]. The most common way to obtain the SPH is by questioning the participant about his or her current or previous health status, offering as possibilities: very bad, bad, fair, good, or very good, within the answers to choose from [16]. Negative SPH states have been found to be associated with increased relative risks of morbidity and death from any cause, compared to positive health states [17].

The field of knowledge demonstrating the multiple health benefits of physical activity (PA) is very broad in the scientific literature [18], considering PA as “body movements generated by skeletal muscle contractions, which cause an elevation above the basal metabolic rate of energy expenditure” [19]. Evidence has been found of reductions in the prevalences of hypertension [20,21], type 2 diabetes [22], mental disorders [6], or different types of cancers [23] in populations that performed PA versus those that were inactive, as well as evidence of being a suitable means of increasing cardiorespiratory fitness (CRF) [24], one of the most important known predictors of morbidity, mortality [25], and health-related quality of life (HRQoL) [26].

Some studies have even found that PA could mitigate the negative effects of smoking [27]. In contrast, physical inactivity, considered as performing an insufficient amount of physical activity, not complying with specific physical activity guidelines [28], has been found to be related to health risk factors [29] and to different pathological conditions and diseases [30,31]. Therefore, physical inactivity could be an enhancer of the negative effects of smoking, increasing the risks of lung cancer [32], or having a negative SPH [33].

For this reason, the objectives of this research were: to analyze the relationships between SPH and the relationship with smoking in the Spanish population; to analyze the relationships between the level of PA and the relationship with smoking in the population, as well as the relationships between the level of PA and SPH health, according to the relationship with smoking in the Spanish population, in the general population, and by sex. In addition to calculating the risks of having a negative SPH in relation to smoking, according to the PA level of the population. It was hypothesized that: performing PA reduces the prevalence of negative SPH in the Spanish smoking population and that physical inactivity increases the risks of presenting a negative HPS in the smoking population compared to performing moderate and/or intense PA.

## 2. Materials and Methods

### 2.1. Ethical Considerations

Approval by an accredited Ethics Committee was not required. The data used for this research were obtained from non-confidential public files of the Spanish National Health Survey 2017, provided by the Ministry of Health, Consumption and Social Welfare (MSCBS), according to Regulation (EU) 2016/679 of the European Parliament and Council of 27 April 2016 on the protection of individuals with regard to the processing of personal data and the free movement of such data.

### 2.2. Study Design

The design of this research consisted of a descriptive cross-sectional study, based on the data obtained in the ENSE 2017, adult questionnaire [34], by interviewing the Spanish population over 15 years of age. The ENSE 2017 [34] was conducted and published by the MSCBS, in collaboration with the National Institute of Statistics (INE). The ENSE 2017 was conducted by accredited interviewers through telephone interviews. The interviews were conducted between October 2016 and October 2017.

### 2.3. Participants

This research had a final sample of 17,708 participants between 15 and 69 years of age: 8482 men and 9226 women. To arrive at this sample, data were taken from the 23,089 people surveyed in the ENSE 2017, excluding those over 70 years of age, as they were not questioned about the PA performed, as well as 60 participants, for not including data in the PA questions, and 9 participants, for not including data on their relationship with tobacco.

To form the initial sample of the ENSE 2017, the selection of participants was carried out using a stratified three-phase random sampling system, considering the Spanish population aged 15–103 years.

### 2.4. Variables and Procedures

From the ENSE 2017 [34], data were used for the variables: Age, Sex, SPH (G21), PA (p. 113, p. 114, p. 115, p. 116, and p. 117), and p. 121 (question referring to the relationship with tobacco).

Derived from the data collected on these variables, the following variables were created:

Age groups: youth (18–34 years. 1), young adults (35–49 years. 2), older adults (50–64 years. 3), and seniors (65–69 years. 4) [35].

Gender group: men (1) and women (2).

SPH groups: based on responses to item G.21 (“In the last twelve months, would you say your health status has been very good, good, fair, fair, bad, very bad?”), groups were created: (1) Negative (grouping participants with “Bad” or “Very bad” responses. (2) Fair (grouping participants with “Fair” responses), and (3) Positive (grouping participants with “Good” or “Very good” responses. This assessment of self-perceived health was proposed by the WHO [36,37] and adopted by the European Health Survey [38] and national health surveys, such as those of Spain, Italy, Greece, and others [36,39,40].

Relationship with tobacco: according to the answers given to item p.121 (Could you say whether you smoke? With possible answers: “Yes, I smoke daily” (1), “Yes, but not daily” (2), “I do not currently smoke, but I have smoked before” (3), “I do not smoke, nor have I smoked regularly” (4), “Don’t know”, and “No answer” (NS/NC). Groups were created: Smokers (1), Occasional smokers (2), Ex-smokers (3), Smokers (2), and Non-smokers (4), depending on the answer given to the question.

Physical activity index (PAI): Index created to quantify the physical activity carried out by the population, taking the participants’ responses to the items: Q.113, Q.114, Q.115, and Q.116; questions from the International Physical Activity Questionnaire (IPAQ) in its Spanish version [41] included in the ENSE 2017. The answers given were assigned a series of factors adapted from the Physical Activity Index [42]. The formula and factors applied for the creation of this index have already been described in previous research [43].

The PAI could take values between 0 and 67.5, then the population was ordered from highest to lowest PAI, and the population’s PAI percentiles were calculated to create the different physical activity levels (PAL).

Physical activity level (PAL). Four PA level groups (PAL) were formed according to the values achieved in the PAI. Participants were grouped into the following PA levels: **inactive** (People with PAI = 0; responded to Q.117 (“Now think about the time you spent walking in the last 7 days”) of the ENSE 2017, “No day more than 10 min in a row”), **walker** (People with PAI = 0; declared in Q.117 of the ENSE 2017, to have walked, at least one day a week more than 10 min in a row), **active** (People with a PAI between 1 and 30, corresponding to the 90th percentile of the population), **very active** (People with a PAI above 30, corresponding to people in a population percentile above the 90th percentile).

### 2.5. Statistical Analysis

The statistical software IBM SPSS Statistics version 25 (IBM Corp., Armonk, NY, USA) was used to perform all statistical procedures.

First, the distribution followed by the variables of interest was tested using the Kolgomorov–Smirnov test. Subsequently, the sample was characterized by means of descriptive analysis, presenting the data with the central values of the population: median and interquartile range (continuous variables) and in absolute and relative frequencies (ordinal variables), performing non-parametric tests to verify the existence of possible inter-group differences between sexes: Mann Whitney U (continuous variables) and z-test for independent proportions (ordinal variables). The chi-square statistic was used to analyze possible dependence relationships between the ordinal variable: SPH with respect to PAL and with the relationship with smoking of the participants in the general population and in the different subgroups. Finally, odds ratios (OR) and relative risks (RR) were calculated, with their respective confidence intervals (95%CI), in addition to Spearman’s *rho*, to carry out a correlation analysis between variables. For all this, a significance level < 0.05 was established.

## 3. Results

Insufficient evidence was found to be able to assume the normality followed by the distributions of the variables of interest after performing the Kolgomorov–Smirnov test.

No statistically significant differences were found in the median age of both sexes; the median age was 47 in the general population and in both sexes (*p* = 0.313 in the Mann–Whitney U-test) (Table 1). Similarly, no dependency relationships were found between the age group and the sex of the participants (*p* = 0.286 in the chi-square test) (Table 1).

**Figure A1:**
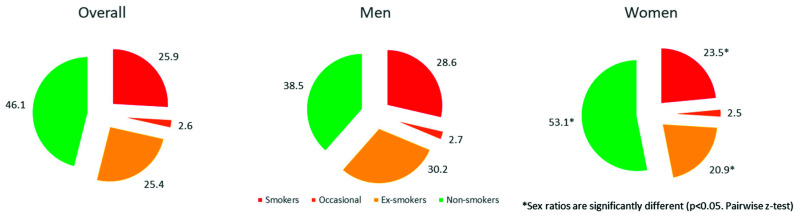
Smoking status in the general Spanish population and sex ratios.

Table 1 shows that in Spain, 46.1% of respondents reported never having smoked, with 53.9% of the population reporting having, or having had, some kind of relationship with tobacco (smokers, occasional smokers, or ex-smokers). In addition, statistically significant differences were found between the type of tobacco use and the sex of the participants (*p* < 0.001 in the chi-square test), with a higher number of male smokers than female smokers (28. 6% vs. 23.5%, *p* < 0.05 in the z-test), more men than women who had quit smoking tobacco (30.2% vs. 20.9%, *p* < 0.05 in the z-test) and a higher number of women than men who had never smoked (53.1% vs. 38.5%, *p* < 0.05 in the z-test) (Appendix A, Figure A1).

**Figure A2:**
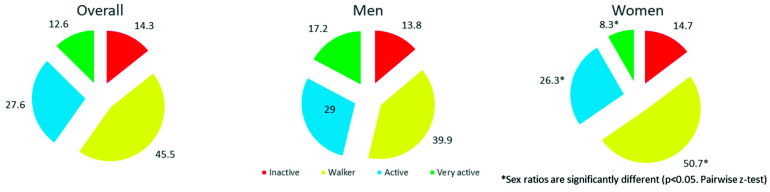
Level of physical activity in the general Spanish population and according to sex.

Regarding PAL and sex, significant differences were found (*p* < 0.001, chi-square test) (Table 1), with women walking more than men (50.7% vs. 39.9%, *p* < 0.05, z-test), and men performing more high/very high level PA than women (17.2% vs. 8.3%, *p* < 0.05, z-test) Appendix A, Figure A2).

With regard to health perception, significant differences were observed in participants’ smoking status, both in the general population and in both sexes (*p* < 0.001, chi-square test). Significant differences were found between the proportions of people with positive SPH (good or very good), according to the relationship they had with smoking (*p* < 0.05, z-test) (Table 2).

**Figure A3:**
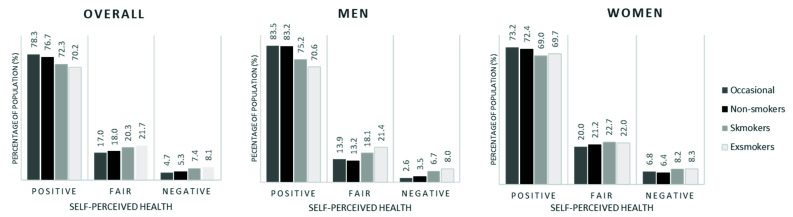
Self-perceived health of the Spanish population, according to their smoking status.

Table 2 shows that the proportion of people with a positive SPH in all groups of smokers and non-smokers was above 70%, while the percentage of respondents with a negative SPH (bad or very bad) was in the range of 8.1–5.7%. Data analysis showed significant differences in proportions of people with a negative SPH between ex-smokers and non-smokers (8.1% vs. 5.3%, *p* < 0.05, z-test). However, they were higher in men (4.5 percentage points) than in women (1.9 points); the prevalence of men and women with negative SPH in ex-smokers showed a difference of 0.3 percentage points, whereas these differences reached 2.9 percentage points between sexes in non-smokers with negative health. Regular SPH status also showed differences in proportions between the different tobacco-related conditions, from 21.7% in Ex-smokers to 17% and 18% in Occasional and Non-smokers. However, these differences were only found in men, being even larger: 8.2 percentage points between Ex-smokers and Non-smokers, being statistically significant differences (*p* < 0.05, z-test). In women, no differences were found in the proportions of Regular HPS in the four groups of smoking relationships, with a prevalence of 21–22% in all cases (Appendix A, Figure A3).

**Figure A4:**
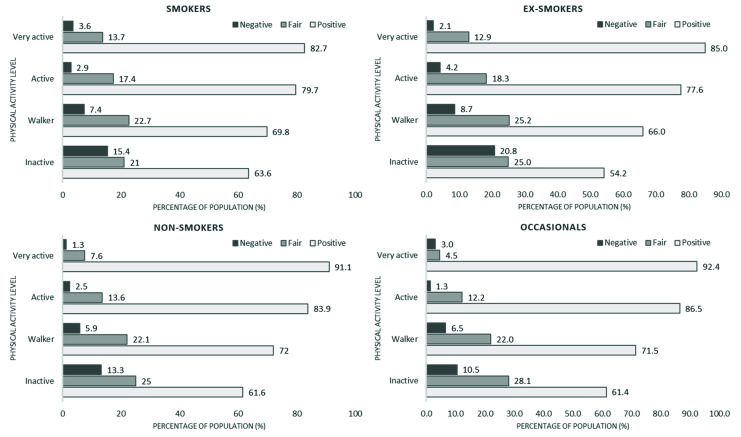
Self-perceived health of the Spanish population, according to their level of physical activity for each smoking status.

Table 3 shows the proportions of SPH according to the participants’ PAL in all tobacco-related conditions. In all tobacco-related conditions, dependence relationships were found between the PAL and the SPH (*p* < 0.001, chi-square test) of the general population. In all cases, the proportions of people with a positive SPH were higher in the higher PALs than in the Inactive and Walkers, showing significant differences (*p* < 0.05, z-test) (Table 3). In the Smoking groups, the proportions of positive SPH increased from 63.6% in Inactives to 82.7% in Very actives, an increase of 19.1 percentage points. Significant differences were found between Inactives and Walkers and between both the Actives and Very Actives (*p* < 0.05, z-test). These differences were as high as 29.5 percentage points in Non-smokers, with similar differences found in all other smoking-related conditions; however, in Ex-smokers and Non-smokers, significant differences were found between the proportions of the four PA groups (*p* < 0.05, z-test). Conversely, the proportions of people with negative SPH were found to be higher in the Inactive than in the higher PALs for all tobacco-related conditions, and these differences were significant (*p* < 0.05, z-test). In Inactive smokers had a proportion of 15.4% compared to 2.9% in Active smokers, a 12.5 points difference (*p* < 0.05, z-test). These differences were as high as 18.7 percentage points between Very Active and Inactive ex-smokers (*p* < 0.05, z-test). SPH Regular also saw reduced proportions of Inactives and Actives in all smoking relationships except Smokers. In contrast, this was true for all smoking relationships between Inactives and Very actives (*p* < 0.05, z-test) (Appendix A, Figure A4).

The odds of having a negative SPH in the Inactive groups were found to be increased in Smokers, Ex-smokers, and Non-smokers relative to the higher PAL groups, from Walkers to Very active (Table 4).

In Smokers, significant ORs and RRs were found between Inactives and the rest of PAL, from Walkers (OR: 2.27. 95%CI: 1.78–2.91. RR: 2.08. 95%CI: 1.67–2.58), the group with the lowest increased risk, versus Actives (OR: 6.02. 95%CI: 3.99–9.07. RR: 5.24. 95%CI: 3.56–7.73), the group with the highest increased risk. These risks were even higher in Ex-smokers (OR: 2.74 in Inactives versus Walkers; OR: 11.98 in Inactives versus Very actives) and Non-smokers (OR: 2.46 in Inactives versus Walkers; OR: 11.43 in Inactives versus Very actives). In addition, weak to moderate correlations were found across the different smoking relationship conditions between PAL and negative health perception or not (Table 4).

Finally, the correlation analysis found that SPH, through Spearman’s *rho*, presented weak and moderate correlations, although statistically significant (*p* < 0.001) between sex (*rho* = −0.068), age group (*rho* = −0.230 in the general population; *rho*: −0. 222 in men; *rho*: −0.226 in women), PAL (*rho* = 0.203 in the general population; *rho*= 0.220 in men; *rho* = 0.186 in women), and smoking relationship (*rho* = −0.065 in the general population; *rho* = −0.123 in men; *rho*: −0.036 in women).

## 4. Discussion

### 4.1. Main Findings and Theoretical Implications

Among the main findings, it was found that among respondents aged 18–69 years living in Spain, 46.1% reported never having smoked, and 53.9% reported having had some kind of relationship with tobacco, with current smokers accounting for 25.9%. This percentage is slightly higher than the 22.3% shown by the WHO in 2020 for the world population that habitually used tobacco, where the percentage of male smokers (36.7%) was clearly higher than that of women (7.8%), a difference that was also shown in our research [44]. Dependence relationships were shown between smoking status and sex of participants, with significantly higher proportions of never-smokers among women than men, in contrast to smokers and ex-smokers, where the proportions were significantly higher among men than women. This finding is consistent with global smoking data from 2015, where the overall prevalence of female smokers (5.4%) in the ten countries with the largest smoking populations was lower than the overall male smoking prevalence (25.0%) [45].

In the present research, we found dependence relationships between PAL and the sex of the participants, with a higher proportion of female walkers than male walkers and a higher proportion of males performing high or very high PAL compared to females, these differences being statistically significant. These results are in line with other studies; for example, Moral-García et al., in a sample of 516 adolescents aged 12 to 16 years, showed that females perform more moderate intensity PA than males, with the male gender being more active and carrying out moderate to vigorous intensity PA [46]. This could be due to the fact that males are more motivated by obtaining immediate results aimed at competition while females tend to seek physical activities with long-term goals [47].

Dependence relationships were found between SPH and smoking status. In the general population, statistically significant differences in proportions were established in men and women, with a higher proportion of occasional smokers and non-smokers than smokers and ex-smokers for SPH positive in the general population and in men (ditto in women, but in occasional smokers, the differences between the proportions were not statistically significant). In the case of negative SPH, a higher proportion of smokers and ex-smokers than occasional smokers or non-smokers were found in the general population and in men (same for women, but in occasional smokers, the differences between proportions were not statistically significant), and these differences were statistically significant.

In the study by AlDukhail et al., a higher negative SPH was also observed in US smokers and ex-smokers aged 18–65 years and older than in people who never smoked, and these percentages were higher than in the present study. This may be due to the fact that the data were collected during the COVID-19 pandemic and also that regular SPH was not taken into account, with people who answered “regular” in their self-assessment of their health being considered SPH negative. However, in their study, the proportions of negative SPH were slightly higher in smokers (21%) than in ex-smokers (19.3%), contrary to our research (7.4% smokers and 8.1% ex-smokers) [48]. The same happened in the study by Ferreira De Sousa et al. in Brazilian adolescents aged 15–19 years; smokers and ex-smokers had higher prevalences of negative SPH than non-smokers, with the percentages of smokers being slightly higher than those of ex-smokers [49].

Females had higher prevalences of negative SPH than males in all four categories of smoking relatedness. This is similar to what happened in other studies, for example, in the case of adolescents aged 14–19 years, where the prevalence of negative SPH was 33% in females compared to 19% in males. The percentages in the study by Silva et al. were higher than in the present study because, in their research, the SPH alternatives were established dichotomously (positive and negative), introducing the “regular” response in the negative SPH, with the percentages of positive SPH being similar to those in the present study [50]. As theorized in other studies, this could be due to an increased concern for their health [51,52] and higher body dissatisfaction in girls, possibly related to the existing media pressure on the female figure, which may have a negative influence on SPH [53,54].

In line with the initial hypothesis, dependent relationships were found between PAL and SPH in the general population according to smoking status. As in other studies, better SPH was associated with higher PAL [55]. In the case of negative SPH, the proportions of inactive were higher than those of active and very active in smokers, ex-smokers, and non-smokers (in occasional smokers, it was similar, but no significant differences were found between the proportions in the case of very active smokers), and these differences were statistically significant. Therefore, our research and other studies show that regular PA could have a positive impact on a person’s SPH [56], even if they suffer from respiratory, musculoskeletal, and other chronic diseases such as cancer and diabetes mellitus [57]. Therefore, being physically active or having a higher PAL could be a key factor in having a more positive PHS in people whose health status is affected by certain pathologies or who have certain conditions that affect their health, as is the case of the smokers in our research.

Finally, our study performed a calculation of the risk of having a negative SPH when the population is smokers. This calculation showed that inactive smokers had a higher risk of having a negative SPH than walkers (OR = 2.27, 95% CI: 1.78 to 2.91). Moreover, the higher the PAL (active and very active), the lower the risk of having a negative SPH (active; OR = 6.02, 95% CI: 3.99–9.07) and (very active; OR = 4.90, 95% CI: 2.91–8.24). However, active subjects were observed to have a similar risk to very active subjects (OR = 0.81, 95% CI: 0.44–1.49). Comparing these negative SPH ORs with ex-smokers and non-smokers, we observe that the results are similar to those of smokers, i.e., being more inactive might increase the risk of having a negative SPH than more active subjects.

These results are in line with those obtained by Piko et al. in their study of adolescents. People who self-perceived their fitness as poor/regular were associated with a higher likelihood of negative/regular SPH (OR = 4.87, 95% CI 3.23–7.33) than people who self-perceived it as good/excellent. For smoking status, regular smokers were associated with a higher likelihood of negative/regular SPH (OR = 2.19, 95% CI 1.40–3.43) than non-smokers [58]. In the case of the Spanish population aged between 15 and 69 years, the ORs are similar to those of the present study, with inactive people being more likely to have a negative SPH than subjects whose PAL is high or very high [39].

In relation to the data reflected in our research, apart from the PAL showing the influence on people’s SPH, smoking status also has some impact on a person’s SPH. In several studies, it was shown that being a smoker or ex-smoker influenced a person’s SPH, with smokers and ex-smokers being more likely to have a negative SPH than people who never smoked in both men and women [17,59].

### 4.2. Practical Implications

Although smoking is associated with poorer SPH, physical inactivity may be an even more important factor, outweighing the negative effects of smoking on population SPH, whereas PA could mitigate these effects. Therefore, moderate and/or intense PA should be recommended for smokers, non-smokers, and ex-smokers alike, if the aim is to improve the population’s SPH. Therefore, both public administrations and health services at the national and regional levels could plan strategic actions to favor a reduction in the prevalence of negative SPH through the promotion of active lifestyles by means of regular PA practice.

In promoting an increase in PAL to reduce the OR of a negative SPH, it would be advisable to invest in health education and training throughout the life cycle. In this sense, it would be interesting to promote multidisciplinary work where professionals from different fields would work in a coordinated manner to achieve this proposal, with sports science professionals being key to prescribing physical exercise adapted to the patient’s conditions.

In relation to the above, lack of time or desire is one of the main reasons for not engaging in PA and the adoption of sedentary behavior [60,61], and this situation may influence SPH and increase the likelihood of it being negative. Therefore, implementing effective and long-lasting PA programs, such as active breaks [62,63] in people’s working or student life, could be an interesting measure to solve this problem and promote a more positive SPH in the individual. Public bodies (companies, institutes, etc.) should include these active breaks, which could reduce the socio-economic and health costs of future or current illnesses.

### 4.3. Limitations and Future Lines

In the present research, we encountered some limitations; for example, we did not carry out a gender differentiation in the study of the association of PA level with the risk of negative SPH. Therefore, as a future line of research, it would be innovative to explore how PA level influences each sex when differences in SPH between men and women are detected.

One of the main limitations of the study was the measurement of SPH from a single question, as Cullati et al. state in their study, the differences in the percentages of the overall variance explained by the way SPH was assessed were not equivalent in their ability to capture the overall health of respondents [64]. Moreover, along these lines, Zajacova et al. report in their study, in which participants reported their SPH on two occasions one month apart, that 40% of respondents changed their health rating between interviews, which they indicated as moderate test-retest reliability [65]. Nevertheless, this form of SPH assessment has been used in a multitude of studies [66,67,68].

Cause–effect relationships could not be established as this was a cross-sectional study based on national health surveys, which is an intrinsic limitation of the study design. Therefore, future research could complement the findings of this study with research designs that can establish causal relationships. For example, carrying out this work longitudinally with objective PA data through public institutions such as the Ministry of Health itself would improve and contrast the results obtained in this survey. The research deals with self-reported PA data, and since it is not objective data, subjects may overestimate or underestimate when measured subjectively. Therefore, future research could use methods and materials conducive to measuring PA objectively.

The type of PA performed by each individual was not taken into account, which may have some influence on the individual’s SPH.

Certain variables were not introduced, such as economic status and level of education, among other socio-demographic, socio-cultural, and socio-economic biases, which could exert some influence on people’s HPS, as has been shown in other studies [69,70,71]. In relation to this, in future research, it would be novel to analyze the influence of PA on the prevalence rates of SPH, dividing the sample by age ranges and socioeconomic status of the participants.

The present study did not take into account whether the participants had comorbidities associated with smoking, i.e., whether they suffered from cardiorespiratory pathologies such as lung cancer, Chronic Obstructive Pulmonary Disease (COPD), etc. These diseases could exert some influence on the individual’s SPH and even on his or her PAL. Therefore, another future line of novel research would be to study the prevalence of SPH and its association with PAL in various pathologies, especially those related to smoking status, such as COPD or other cardiorespiratory diseases.

## 5. Conclusions

The population groups of current and former smokers in Spain had worse SPH than non-smokers. Habitual or former smoking was associated with a higher prevalence of people with negative SPH in Spain, both in the general population and in both sexes.

The relationship with tobacco in the population showed a relationship of dependence on the PAL. There is a higher prevalence of smokers and ex-smokers in the inactive population groups or those who only walk, compared to those with a higher PAL, including moderate and/or intense activities, which is found both in the general population and in both sexes.

SPH was related to PAL in all population groups, depending on their relationship to smoking. Belonging to the Inactive group was related to a higher prevalence of negative SPH versus Walking or moderate and/or intense PA in smokers, ex-smokers, and non-smokers. Although walking was associated with a reduction in prevalence compared to the Inactive group, this reduction is smaller than in the higher PALs. Conversely, the Active or Very Active groups were associated with higher prevalences of positive SPH, being higher than the prevalences of positive SPH of Walkers, and these, in turn, were higher than the prevalences of Inactive.

Physical inactivity increases the risks of having a negative SPH, regardless of the relationship with smoking, compared to walking or higher PALs. Walking may be sufficient to reduce the odds of having a negative HPS in inactive populations. However, walking increases the risk compared to moderate and/or intense PA populations.

## Figures and Tables

**Table 1:** Socio-demographic characteristics, smoking status, and physical activity level of the Spanish population aged 18–69 years in 2017.

Variables				
Age (Years)	Overall = 17,708	Men = 8482	Women = 9226	*p*
Median (IQR)	47 (21)	47 (21)	47 (21)	0.313
Mean (SD)	45.8 (14.1)	45.7 (14.1)	46.0 (14.1)	-
Age group	Overall n (%)	Men n (%)	Women n (%)	*p* *
18–34 years	3871 (21.9)	1851 (21.8)	2020 (21.9)	0.286
35–49 years	6174 (34.9)	2990 (35.3)	3184 (34.5)	
50–64 years	5953 (33.6)	2858 (33.7)	3095 (33.5)	
65–69 years	1710 (9.7)	783 (9.2)	927(9.7)	
Smoking group	Overall n (%)	Men n (%)	Women n (%)	*p* *
Smokers	4590 (25.9)	2425 (28.6)	2165 (23.5) b	<0.001
Occasionals	465 (2.6)	230 (2.7)	235 (2.5)	
Ex-smokers	4489 (25.4)	2564 (30.2)	1925 (20.9) b	
Non-smokers	8164 (46.1)	3263 (38.5)	4901 (53.1) b	
PAL	Overall n (%)	Men n (%)	Women n (%)	*p* *
Inactive	2532 (14.3)	1174 (13.8)	1358 (14.7)	<0.001
Walker	8062 (45.5)	3385 (39.9)	4677 (50.7) b	
Active	4886 (27.6)	2460 (29.0)	2426 (26.3) b	
Very active	2228 (12.6)	1463 (17.2)	765 (8.3) b	

IQR (Interquartile range); SD (Standard deviation); n (Number of participants); % (Percentage); PAL (Physical activity level); Inactive (PAI = 0; Report not walking for more than 10 min at a time, no day on the week). Walkers (PAI = 0; Report walking more than 10 min in a row, at least one day on the week); Active (PAI between 1 and 30). Very active (PAI > 30); *p* (*p*-value from Mann–Whitney U test); *p* * (*p*-value from chi-square test); Smokers (people who report smoking every day); Occasional (report not smoking daily); Ex-smokers (report having smoked, not currently); Non-smokers (report not smoking, nor having smoked regularly); b (Significant differences between sex ratios with *p* < 0.05 at the z-test).

**Table 2:** Relationship between smoking and self-perceived health in the Spanish population, by gender.

Overall					
SPH	Ex-Smokers	Smokers	Occasionals	Non-Smokers	*p*
Positive	3153 (70.2) a	3317 (72.3) b	364 (78.3) c	6264 (76.7) c	<0.001
Fair	972 (21.7) a	932 (20.3) ab	79 (17.0) bc	1469 (18.0) c	
Negative	364 (8.1) a	341 (7.4) a	22 (4.7) b	431 (5.3) b	
**Men**					
**SPH**	**Ex-smokers**	**Smokers**	**Occasionals**	**Non-smokers**	***p***
Positive	1811 (70.6) a	1823 (75.2) b	192 (83.5) c	2716 (83.2) c	<0.001
Fair	548 (21.4) a	440 (18.1) b	32 (13.9) bc	432 (13.2) c	
Negative	205 (8.0) a	162 (6.7) a	6 (2.6) b	115 (3.5) b	
**Women**					
**SPH**	**Ex-smokers**	**Smokers**	**Occasionals**	**Non-smokers**	***p***
Positive	1342 (69.7) a	1494 (69.0) a	172 (73.2) ab	3548 (72.4) b	<0.001
Fair	424 (22.0) a	492 (22.7) a	47 (20.0) a	1037 (21.2) a	
Negative	159 (8.3) a	179 (8.2) a	16 (6.8) ab	316 (6.4) b	

Data shown in absolute and relative frequencies; SPH (Self-perceived health); Inactive (PAI = 0; People who report not walking more than 10 min at a time, no day of the week). Walkers (PAI = 0; People who report walking more than 10 min in a row, at least one day a week); Active (PAI between 1 and 30); Very active (PAI > 30); *p* (*p*-value from chi-square test); Smokers (people who report smoking every day); Occasionals (report not smoking every day); Ex-smokers (report having smoked, not currently); Non-smokers (report not smoking, nor have smoked regularly); a,b,c (Different subscripts indicate the existence of differences between the proportions of people with different levels of self-perceived health, according to the relationship with smoking, at the 0.05 level in the z-test).

**Table 3:** Relationships between level of physical activity and self-perceived health, according to the relationship with smoking in the Spanish population of the ENSE 2017.

**Smokers**					**Occasionals**				
**Self-Perceived Health**	**Positive**	**Fair**	**Negative**	***p***	**Self-Perceived Health**	**Positive**	**Fair**	**Negative**	***p***
Inactive	507 (63.6) a	167 (21.0) a,b	123 (15.4) a	<0.001	Inactive	35 (61.4) a	16 (28.1) a	6 (10.5) a	<0.001
Walker	1607 (69.8) b	523 (22.7) b	171 (7.4) b		Walker	133 (71.5) a	41 (22.0) a	12 (6.5) a	
Active	812 (79.7) c	177 (17.4) a,c	30 (2.9) c		Active	135 (86.5) b	19 (12.2) b	2 (1.3) b	
Very active	391 (82.7) c	65 (13.7) c	17 (3.6) c		Very active	61 (92.4) b	3 (4.5) b	2 (3.0) a, b	
**Ex-smokers**					**Non-smokers**				
**Self-Perceived Health**	**Positive**	**Fair**	**Negative**	***p***	**Self-Perceived Health**	**Positive**	**Fair**	**Negative**	***p***
Inactive	308 (54.2) a	142 (25.0) a	118 (20.8) a	<0.001	Inactive	684 (61.6) a	278 (25.0) a	148 (13.3) a	<0.001
Walker	1360 (66.0) b	520 (25.2) a	180 (8.7) b		Walker	2531 (72.0) b	777 (22.1) b	207 (5.9) b	
Active	1009 (77.6) c	238 (18.3) b	54 (4.2) c		Active	2021 (83.9) c	328 (13.6) c	61 (2.5) c	
Very active	476 (85.0) d	72 (12.9) c	12 (2.1) d		Very active	1028 (91.1) d	86 (7.6) d	15 (1.3) d	

Data presented in absolute and relative frequencies; Positive (Reporting good or very good health); Fair (Reporting fair health); Negative (Reporting poor or very poor health); PAI (Physical Activity Index); Inactive (People with PAI = 0; Reporting no walking, at least one day a week, 10 min or more in a row); Walkers (People with PAI = 0; Report walking, at least one day a week, 10 min or more in a row); Low/Medium (People with PAI between 1–30); High/Very High (People with PAI > 30); a,b,c,d (Each subscript indicates no difference between the proportions between rows in a column at the 0.05 level in Levene’s test).

**Table 4:** Odds ratio and relative risk of perceiving negative health by physical activity level in the Spanish population in smokers, ex-smokers, and non-smokers in the Spanish population of the ENSE 2017.

**Risks of Negative SPH: Smokers**							
**PAL**		**OR**	**CI 95%**	**RR**	**CI 95%**	***rho***	***p***
Inactive	Walker	2.27	1.78–2.91	2.08	1.67–2.58	0.119	<0.001
	Active	6.02	3.99–9.07	5.24	3.56–7.73	0.223	<0.001
	Very active	4.90	2.91–8.24	4.29	2.62–7.04	0.183	<0.001
Walker	Active	2.65	1.78–3.93	2.52	1.73–3.69	0.087	<0.001
	Very active	2.15	1.30–3.58	2.07	1.27–3.37	0.057	<0.005
Active	Very active	0.81	0.44–1.49	0.82	0.46–1.47	−0.017	0.504
**Risks of negative SPH: Ex-smokers**							
**PAL**		**OR**	**CI 95%**	**RR**	**CI 95%**	***rho***	***p***
Inactive	Walker	2.74	2.13–3.53	2.38	1.92–2.94	0.156	<0.001
	Active	6.05	4.31–8.50	5.00	3.68–6.80	0.265	<0.001
	Very active	11.98	6.53–21.97	9.70	5.42–17.36	0.292	<0.001
Walker	Active	2.21	1.62–3.03	2.11	1.57–2.83	0.088	<0.001
	Very active	4.37	2.42–7.90	4.08	2.29–7.26	0.104	<0.001
Active	Very active	1.98	1.05–3.73	1.94	1.04–3.59	0.05	<0.05
**Risks of negative SPH: Non-smokers**							
**PAL**		**OR**	**CI 95%**	**RR**	**CI 95%**	***rho***	***p***
Inactive	Walker	2.46	1.97–3.07	2.26	1.85–2.77	0.119	<0.001
	Active	5.92	3.94–7.04	5.27	3.94–7.04	0.212	<0.001
	Very active	11.43	6.67–19.57	10.04	5.94–16.96	0.231	<0.001
Walker	Active	2.41	1.80–3.22	2.33	1.76–3.08	0.079	<0.001
	Very active	4.65	2.74–7.88	4.43	2.64–7.45	0.092	<0.001
Active	Very active	1.93	1.09–3.41	1.91	1.09–3.34	0.039	<0.05

OR (Odds ratio); CI 95% (Confidence Interval); RR (Relative Risk); PAI (Physical Activity Index. Scores: 0–67.5); Inactive (PAI = 0; Report not walking more than 10 min at a time on any day of the week). Walker (PAI = 0; Report walking more than 10 min in a row, at least one day a week). Active (PAI between 1 and 30). Very active (PAI between +30); *rho* (Spearman correlation coefficient); *p* (*p*-value).

## Data Availability

Datasets will be available under reasonable request.
